# Cell-Based Therapy for Silicosis

**DOI:** 10.1155/2016/5091838

**Published:** 2016-03-15

**Authors:** Miquéias Lopes-Pacheco, Elga Bandeira, Marcelo M. Morales

**Affiliations:** Laboratory of Cellular and Molecular Physiology, Institute of Biophysics Carlos Chagas Filho, Federal University of Rio de Janeiro, Rio de Janeiro, RJ, Brazil

## Abstract

Silicosis is the most common pneumoconiosis globally, with higher prevalence and incidence in developing countries. To date, there is no effective treatment to halt or reverse the disease progression caused by silica-induced lung injury. Significant advances have to be made in order to reduce morbidity and mortality related to silicosis. In this review, we have highlighted the main mechanisms of action that cause lung damage by silica particles and summarized the data concerning the therapeutic promise of cell-based therapy for silicosis.

## 1. Introduction

The industrialization process steadily increased the occupational exposition of workers to breathable particles in many work environments. Among these particles, silicon dioxide, or silica, has an important role in respiratory occupational diseases' burden. Inhalation of silica particles causes silicosis: a persistent inflammation with granuloma formation that leads to tissue remodeling and impairment of lung function [1]. Tuberculosis, chronic obstructive pulmonary diseases, and rheumatoid arthritis are some common complications associated to silicosis [2–4]. Long-term silica exposure can also cause lung cancer [5, 6]. To date, the available management of silicosis is focused on controlling associated symptoms and comorbidities and no therapy halts or reverses the disease progression. In the end-stage of lung illness, the patient succumbs to death due to respiratory failure [1].

Over the past decades, many efforts have been made to prevent inhalation of silica dust by workers; however, silicosis is still a public health concern worldwide with higher prevalence in developing countries [7]. In Brazil, Holanda and collaborators showed a prevalence of silicosis of 33% of pit diggers in Ceará state [8] and over 4,500 cases of silicosis cases were related to gold-miners between 1978 and 1988 in Minas Gerais state [9]. Currently, it is expected that over 6 million workers are daily exposed to silica in various labor fields nationwide [4, 10]. China reported more than 500,000 silicosis patients between 1991 and 1995 with about 6,000 new cases and over 24,000 deaths per year [1]. In India, 10 million workers are exposed to silica dust with high risk of developing the disease [11].

Silicosis is also an occupational health issue in developed countries. More than 3 million workers were exposed to silica particles between 1990 and 1993 in Europe, 600,000 of them being in the United Kingdom [12]. In the United States, more than 100,000 workers were exposed to silica dust and over 3,500 new cases were related per year from 1987 to 1996 [13]. The implementation of protective measures has a declined number of new silicosis cases and the mortality rate. However, silicosis is still incurable and new outbreaks happen, such as the exposure of quartz conglomerate workers in Spain, in which the median age of the cohort was 33 years [14]. Furthermore, there is an expansion in the number of work environments with potential silica dust exposure, such as jeans sandblasting and quartz.

An incremental number of articles have shown the efficacy of either systemic or intratracheal administration of stem cells in several animal models of lung injury [15]. Among those, the bone marrow cells are the most studied. They were able to promote lung parenchyma reepithelization, modulate the immune response, and decrease the tissue remodeling [16–19]. Silicosis has a unique pathogenesis process, in which the phagocytosis and release of silica particles in the lung tissue drive the disease progression [20]. This review highlights the main mechanisms of action of silica dust in the alveolar environment and how cell therapy may help patients with silicosis by modulating the inflammation, reducing fibrosis, and, thus, improving lung function.

## 2. Pathogenesis: The Cycle of Damage in Lung Tissue

Silica particles that overcome the mucociliary defense mechanism in the airways and reach the distal portions in the lung begin the pathogenesis cascade. The most pathogenic particles for humans are those under 10 *μ*m, since they have the aerodynamics required to reach bronchioles and alveoli [4, 21].

**Figure 1:**
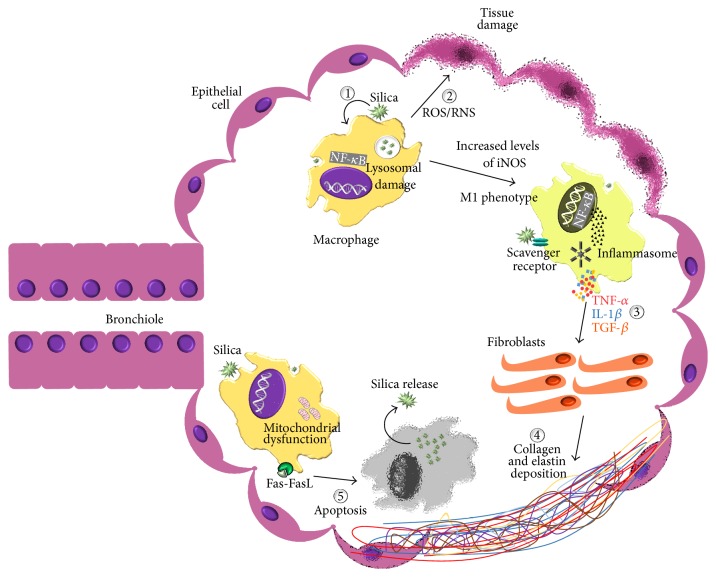
Silicosis pathogenesis. The main mechanisms that orchestrate the disease progression caused by silica-induced lung damage are (1) direct cytotoxicity, (2) production of reactive oxygen species (ROS) and reactive nitrogen species (RNS), (3) secretion of inflammatory and fibrotic mediators, (4) lung remodeling through collagen and elastin deposition, and (5) cell death by apoptosis.

Silicosis is orchestrated by a cycle of phagocytosis and release of these particles that lead to epithelial damage, lung remodeling, and reduction of gas exchange area. Silica-induced lung damage occurs by five main mechanisms (Figure 1): (1) direct cytotoxicity, (2) generation of reactive species, (3) production of cytokines and chemokines, (4) fibrosis, and (5) cell death by apoptosis [22–25].

Silica particles present a piezoelectric property, which means that under certain applied pressure, the crystals acquire electrical polarity. This provides the ability of silica dust to cause direct cytotoxicity. The particles react with resident cells and cause lipid peroxidation of the membrane in bronchoalveolar cells. Newly fractured particles are the most cytotoxic, since they can generate more free radicals in aqueous medium that disrupt the plasma membrane and release lysosomal enzymes, thus causing tissue damage [22, 26].

Reactive oxygen species (ROS) and reactive nitrogen species (RNS) are highly reactive chemicals often produced for the biological defense system against noxious agents. However, the interaction between alveolar macrophages and silica particles causes respiratory burst with high consumption of oxygen, increased levels of inducible nitric oxide synthetize (iNOS), and production of ROS, which is damaging to lung cells [27, 28]. Among ROS/RNS, nitric oxide has a critical role in silicosis pathogenesis. Nitric oxide is formed from the conversion of the amino acid L-arginine to L-citrulline in presence of iNOS, which then interacts with superoxide and forms peroxynitrite that damages mitochondria and DNA, and inactivates several proteins [29–31].

The oxidative stress activates transcriptional factors, such as the nuclear factor kappa B (NF-*κ*B) [31] and activator protein 1 (AP-1) [32]. The interaction of silica with macrophages and epithelial cells translocates NF-*κ*B from the cytoplasm to the nucleus, where it binds to the DNA, starting the transcription and translation of genes involved in inflammatory and fibrogenic processes. The release of cytokines, chemokines, lipid mediators, and growth factors recruits polymorphonuclear and mononuclear cells to the alveolar spaces and around the silica particles, which contribute for the formation of granulomas [22, 31, 33].

Leukotriene B4 is a lipid mediator that mastocytes produce in response to silica stimulation. The leukotriene B4 increases the amount of neutrophils in the inflammation site, and it is also involved in tumorigenesis [34]. Furthermore, the macrophage inflammatory protein- (MIP-) 1 and MIP-2 are chemokines that increase the number of macrophages in response to silica-induced lung injury [29, 35]. Macrophages are the first cells to interact with silica particles and this interaction can activate a range of extracellular signals that lead to polarization of these cells [35]. The M1 macrophages are responsible for the antimicrobial and inflammatory responses, and cells are polarized to this phenotype in the presence of iNOS. Macrophages polarize to M2 phenotype in response to arginase, and in this case they are involved in the inflammation resolution and tissue repairs [36–38].

In alveolar macrophages, the scavenger receptors- (SR-) A and MARCO (macrophage receptor with collagenous structure) recognize and phagocyte the particles to remove them from alveoli [24, 38]. Nevertheless, knockout of MARCO increases inflammation after silica exposure in mice, due to increased lysosomal membrane permeabilization and inflammasome activation [39]. Once silica activates the NLRP3 (NOD-like receptor, pyrin domain containing 3) inflammasome, the cleavage of caspase-1 and production of cytokines occur, such as interleukin- (IL-) 18 and IL-1*β* [39, 40].

During silica-induced inflammation, epithelial cells and alveolar macrophages secrete IL-1*α* and IL-1*β*, respectively [26, 41]. Both cytokines are involved in fibroblasts activation and collagen deposition. IL-1*α* and IL-1*β* are agonists, while the IL-1 receptor antagonist (IL-1Ra) occurs naturally in response to inflammation and it can inhibit the effects of IL-1 [42, 43]. Furthermore, IL-1*β* and tumor necrosis factor- (TNF-) *α* increase the expression of IL-6, another mediator involved in the disease progression [44, 45].

The increased expression of TNF-*α* during silicosis leads to fibroblasts recruitment and proliferation [31]. TNF-*α* also can connect to the cell death receptor and start the apoptosis cascade. The knockout to Fas ligand (FasL) in mice and the use of anti-TNF antibody were able to prevent the silica-induced lung injury [46, 47].

Once fibroblasts were recruited to the damage site, the transforming growth factor- (TGF-) *β* induces the collagen deposition [48], as well as increased elastin production [49]. The increased expression of metalloproteases- (MMP-) 2 and MMP-9 and inhibition of tissue inhibitor of metalloprotease- (TIMP-) 1 and TIMP-2 cause a lung parenchyma restructuration on the course of the disease [50, 51]. The increased tissue damage caused by silica particles, the degradation of extracellular matrix by MMP, and the exacerbation of concentric deposition of collagen are responsible for granuloma formation and lung remodeling that impairs lung function.

The process of apoptosis is a result of the mitochondrial dysfunction and increased expression of death receptors and their ligands, such as FasL and TNF [46, 47, 52]. Besides the oxidative stress, the silica particles lead to loss of mitochondrial membrane potential, which is followed by activation of caspase-9 and caspase-3 and DNA fragmentation [25]. Cells release chemotactic factors during apoptosis that recruit new inflammatory cells, increasing the inflammation. Importantly, macrophages undergoing apoptosis also release silica particles back to the lung parenchyma, where they are phagocyted again by other macrophages, perpetuating the tissue damage cycle [27].

## 3. Silicosis Treatment: Still an Unmet Need

Despite the fair amount of outrage over the epidemiologic persistence of silicosis, little has been achieved in terms of treatment development. Research towards a satisfactory therapy for silicosis is happening in a much slower pace than for other chronic lung diseases. Only two registered clinical trials have been concluded in the past 10 years on silicosis treatments [53, 54], leaving a gap to be filled specially by developing countries, such as Brazil, India, and China, in which the prevalence of silicosis facilitates subject availability for clinical tests.

Management of silicosis consists of the use of bronchodilators/cough medication, prevention of exposure to irritants, and close monitoring for respiratory infections. Corticosteroid therapy may be used to reduce bronchitis and ameliorate symptoms as a short-term treatment, but its long-term positive effects have not been proved; it has the disadvantage of increasing risk of infections [55, 56]. Aluminum-based compounds were extensively studied for their ability to coat silica particles, reducing crystals reactivity and therefore protecting the lung tissue. Nevertheless, despite of good results achieved in experimental models [57], clinical studies showed no efficacy or sustained effects of treatment with aluminum in humans [58, 59]. Polyvinyl-pyridine-N-oxide (PVNO), a polymer able to promote cytoprotective effects in* in vivo* and* in vitro* models of silica-induced fibrosis, was also widely tested. Prophylactic and therapeutic use of PVNO yielded positive results in animal models [60] but had little efficacy in humans. PVNO delayed the progression of fibrosis in patients in one small clinical study but did not alter the outcome three years after treatment [61] and had limited therapeutic effect depending on factors, such as age and severity of the disease in another [62]. Another drug that has been tested for silicosis treatment is the herbal alkaloid tetrandrine. It has been historically employed for the treatment of pneumoconiosis in Chinese medicine, and its therapeutic use for silicosis has been approved by the State Drugs Administration of China. Tetrandrine alone and in combination with other drugs has been tested in animals and in humans [63]. However, robust clinical trials with objective criteria are still needed to prove its clinical efficacy.

Because of the prominent role of IL-1*β* in the pathophysiology of silicosis, targeting this cytokine could be an interesting option of treatment for silicosis. In experimental models, inhibition of IL-1 resulted in reduced expression of TGF-*β*1, collagen I, and fibronectin in the lung [64] and also reduced fibrosis and inflammation in kidneys and heart [65]. Use of an IL-1 receptor antagonist also reduced fibrosis and size of fibrotic nodules in mice [66]. A single report of using the drug anakinra—an IL-1 receptor antagonist—showed amelioration of respiratory symptoms and noticeable improvement in radiology images after six months of treatment in a 37-year-old man [67].

Whole lung lavage seems to have a positive impact in management of silicosis, improving patients prognosis and reducing lung infiltration, though there is no evidence that this approach suffices to prevent disease progression [68–70].

Other approaches have been tested in experimental models: the use of interferon gamma [71], ascorbic acid [72], beta-aminopropionitrile [73], relaxin [74], suppressive oligodeoxynucleotides [75], methyl palmitate [76], N-acetyl-cysteine [77], Dasatinib [78] and Nintedanib [79], and the antagonism/blockage of IL-13 [80] and IL-17A [81], as well as the use of the micro RNA miR-486-5p [82] and gene therapy with a short hairpin RNA to silence *β*-catenin [83]. All of these exerted positive effects in animal models, especially reducing lung fibrosis, were not translated to clinical trials so far.

As a last resource, lung transplantation is indicated to patients with no more pharmacological options. Nevertheless, the long-term survival after transplantation has a poor prognosis [84].

## 4. Cell-Based Therapy: From Bench to Bedside

The first bone marrow transplantation was performed in 1962, in order to use the recently discovered hematopoietic stem cells to restore proper physiology to the bone marrow of a patient [85].

In 1981, embryonic stem cells were described by Evans and Kaufman as pluripotent cells derived from the internal mass of a murine embryo, changing the paradigm for stem cells potential [86]. More recently, stem cells and stem-like cells were found in almost all adult tissues [87]. Cell transplantation began to be thought as a possible treatment for multiple diseases, due to the potential of stem cells to differentiate, replacing damaged cells in target tissues. Moreover, cell transplantation exerted beneficial effects in multiple models of diseases independently of cell homing or differentiation, evidencing a paracrine/endocrine effect of the cells [88, 89].

Cell-based therapies have the advantages of modulating inflammation and affecting the remodeling process concomitantly, without presenting toxicity or immunosuppression. These properties make cell therapy an exceptionally advantageous therapeutic approach for fibrotic lung diseases, including silicosis. Several preclinical studies have been exploiting this approach for the treatment of silicosis (Table 1).

The most extensively studied adult cell source for cell therapy is the bone marrow. The bone marrow contains a multitude of cells in different stages of differentiation. Among those cells, hematopoietic stem cells are of particular importance for their capacity to differentiate into immune cells and to modulate immune cell proliferation and activity [90]. Notwithstanding, in the pool of cells present in bone marrow cells that have a primary role in the maintenance, promoting growth and survival of other cells might have even stronger therapeutic potential. These cells are called mesenchymal stromal cells, and beyond their stromal properties they are also multipotent [91]. Both the pool of mononuclear cells present in the bone marrow and the stromal cells only have been shown to cause improvement in a wide range of inflammatory illnesses. Moreover, cell therapy has yielded positive effects in models of lung fibrotic diseases such as asthma, COPD, and bleomycin-induced lung fibrosis [92–94]. Clinical trials on cell therapy for lung diseases are advancing, and studies have been registered in Europe, Brazil, Australia, Canada, and the United States [95].

In 2009, our group published the first work using cell-based therapy in murine model of silicosis. Lassance et al. used a local infusion of a population of adherent mononuclear cells (BMDC) and evaluated the effects in two time points. The results showed reduction in the inflammation process thirty days after treatment, thus improving lung function; but these beneficial effects seemed to fade within sixty days [96]. A follow-up on this study was published in 2013, showing that multiple doses of BMDC prevent the disease progression. Two infusions of bone marrow cells reduced inflammation (fractional area of granuloma and number of total and M1 macrophages), lung remodeling (TGF-*β* level, deposition of collagen, and elastic fibers), and apoptosis (caspase-3 level and number of apoptotic cells). All these effects result in improvement of lung mechanics parameters. An increased level of IL-1Ra seems to have a role in the sustained cell therapy effects for a longer time in the same model of silicosis [36].

In 2011, a study using the whole pool of bone marrow-derived mononuclear cells (BMMC) in mice showed prophylactic properties on silica-induced lung damage. The systemic infusion of BMMC reduced the mRNA expression of caspase-3, IL-1*β*, IL-1*α*, and TGF-*β* [97]. Furthermore, therapeutic treatment with BMMC in late stages of silicosis was able to reduce lung fibrosis and improve lung function but did not succeed in reversing the inflammation. A reduction in the number of macrophages was accompanied by an expansion of T regulatory cells, which maintained the cellular infiltration, although switching to a different inflammatory profile [16]. BMMC offer the advantages of autologous transplantation, which minimize the possibility of rejection and harvesting and infusion at the same day, without* in vitro *culture expansion.

These results encouraged us to advance to a phase I clinical trial to test the safety of the autologous transplantation of bone marrow mononuclear cells in silicotic patients. Five patients underwent BMMC instillation through bronchoscopy. No adverse effects were observed in these patients up to a year after treatment. Additionally, an early increase in lung perfusion was observed after treatment [54].

The disadvantages of the use of bone marrow mononuclear cells are the invasiveness of the procedure—especially considering that multiple doses may be required for sustained effects—and the variability of the constitution of the bone marrow. Chronic inflammatory diseases can affect the proportions of bone marrow cell populations [101, 102], making autologous transplantation possibly heterogenic over different patients and stages of the disease.

As an alternative to that, mesenchymal cells could constitute an interesting option for cell therapy in silicosis. Zhao and collaborators showed that bone marrow-derived mesenchymal cells (BMSC) promote amelioration of fibrosis and inflammation in a rat model of silicosis, with possible similar mechanisms to those associated with BMMC therapy: suppression of IL1 signaling through IL1-Ra and decrease in TNF-*α* expression [99].

Results from a clinical trial using mesenchymal stromal cells and a genetically modified culture of mesenchymal cells were recently published [53]. In this study, the administration of MSC did not cause any adverse reactions, and after six months oxyhemoglobin saturation in the blood of the patients suggests improvement of gas exchange in the lungs. In some of the patients, a decrease in silica nodules' numbers was also observed.

Another view on the mechanisms of action of mesenchymal cell therapy in silica-induced lung fibrosis has been proposed by Choi et al. in 2014. The authors suggest that extracellular vesicles released by BMSC are vectors of the therapeutic effect and could be used as a treatment by themselves [100].

Additionally, cell therapy with embryonic cells, as a bioengineering approach for the reconstitution of injured epithelium has been investigated in a model of silica-induced injury and was able to prevent fibrosis and decrease mortality [98].

## 5. Conclusion

In view of the lack of effective therapeutic interventions for silicosis, cell-based therapy constitutes a promising treatment for silicosis. The key effects of this therapy include the decrease of deleterious proinflammatory and profibrotic processes, reduction of apoptosis, and enhancement of repair following lung damage. These beneficial effects appear to be independent of cell engraftment, but due to paracrine/endocrine action (e.g., secretion of anti-inflammatory, antifibrotic mediators, and extracellular vesicles). Considering the wide range of cell therapy options, optimization of cell therapy protocols and advancements in clinical trials can lead to an important breakthrough for the management of the patients affected by this persistent disease.

## Figures and Tables

**Table 1:** Experimental studies using cell therapy in silicosis.

Model	Cell type	Route	Number of cells	Follow-up	Reference
C57BL/6	BMDC	i.t.	2 × 10^6^	30 and 60 days	[96]
C57BL/6	BMMC	i.v.	2 × 10^6^	15 days	[97]
Nude mice	HUES-3	i.t.	2.5 × 10^6^	15 days	[98]
Sprague-Dawley	BMSC	i.t.	3 × 10^6^	14 days	[99]
C57BL/6	BMDC	i.t.	2 doses of 2 × 10^6^	60 days	[36]
C57BL/6	BMSC	i.v.	2 × 10^5^	15 and 30 days	[100]
C57BL/6	BMMC	i.v.	1 × 10^6^	70 days	[16]

BMDC: bone marrow-derived cells; BMMC: bone marrow mononuclear cells; BMSC: bone marrow-mesenchymal stromal cells; HUES: human embryonic stem cells; i.t.: intratracheal; i.v.: intravenous.
